# The provision of NHS health checks in a community setting: an ethnographic account

**DOI:** 10.1186/s12913-015-1209-1

**Published:** 2015-12-10

**Authors:** Ruth Riley, Nikki Coghill, Alan Montgomery, Gene Feder, Jeremy Horwood

**Affiliations:** The Centre for Academic Primary Care, School of Social and Community Medicine, University of Bristol, Bristol, BS8 2PS UK; Nottingham Clinical Trials Unit, Faculty of Medicine & Health Sciences, University of Nottingham, Nottingham, NG7 2UH UK; The National Institute for Health Research Collaboration for Leadership in Applied Health Research and Care West (NIHR CLAHRC West), University Hospitals Bristol NHS Foundation Trust, Bristol, UK

**Keywords:** Cardiovascular disease, Black and minority ethnic populations, Health inequalities, Social capital, Ethnography

## Abstract

**Background:**

The UK National Health Service Health Checks programme aims to reduce avoidable cardiovascular deaths, disability and health inequalities in England. However, due to the reported lower uptake of screening in specific black and minority ethnic communities who are recognised as being more at risk of cardiovascular disease, there are concerns that NHS Health Checks may increase inequalities in health. This study aimed to examine the feasibility and acceptability of community outreach NHS Health Checks targeted at the Afro-Caribbean community.

**Methods:**

This paper reports findings from an ethnographic study including direct observation of four outreach events in four different community venues in inner-city Bristol, England and follow up semi-structured interviews with attendees (*n =* 16) and staff (*n =* 4). Interviews and field notes were transcribed, anonymized and analysed thematically using a process of constant comparison.

**Results:**

Analysis revealed the value of community assets (community engagement workers, churches, and community centres) to publicise the event and engage community members. People were motivated to attend for preventative reasons, often prompted by familial experience of cardiovascular disease. Attendees valued outreach NHS Health Checks, reinforcing or prompting some to make healthy lifestyle changes. The NHS Health Check provided an opportunity for attendees to raise other health concerns with health staff and to discuss their test results with peers. For some participants, the communication of test results, risk and lifestyle information was confusing and unwelcome. The findings additionally highlight the need to ensure community venues are fit for purpose in terms of assuring confidentiality.

**Conclusions:**

Outreach events provide evidence of how local health partnerships (family practice staff and health trainers) and community assets, including informal networks, can enhance the delivery of outreach NHS Health Checks and in promoting the health of targeted communities. To deliver NHS Health Checks effectively, the location and timing of events needs to be carefully considered and staff need to be provided with the appropriate training to ensure patients are supported and enabled to make lifestyle changes.

**Electronic supplementary material:**

The online version of this article (doi:10.1186/s12913-015-1209-1) contains supplementary material, which is available to authorized users.

## Background

Cardiovascular disease (CVD) is one of the leading causes of morbidity and premature mortality in the UK [1]. In 2009 the UK National Health Service (NHS) in England introduced the NHS Health Check programme to identify and prevent cardiovascular risk for persons aged 40–74 years who are not on a relevant disease register [2]. The main aim of NHS Health Checks is to identify those at risk of developing heart disease, stroke, diabetes and kidney disease through identifying and modifying behavioural (such as smoking, diet, exercise, alcohol) and physiological (such as high blood pressure or cholesterol) risk factors [2]. Attendees receive a low (<10 %), medium (>10 % <20 %) or high (>20 %) 10 year cardiovascular (QRisk2) score which is based on family history, physiological measurements, socio-economic status and ethnicity [3]. NHS Health checks are funded by the Department of Health via directors of public health in local authorities [4]. NHS Health Checks are usually delivered in family practices by nurses, healthcare assistants, with particpating practices receiving payment for inviting patients and completing NHS Health Checks [1].

The NHS Health Checks are part of a wider public health strategy to improve the health and wellbeing of the nation and reduce inequalities in health [1, 2]. However, critics have questioned the aims of the NHS Health Checks programme, the evidence of its  clinical or cost-effectiveness and the potential for a general health check to reduce morbidity or mortality [5–7]. The Royal College of General Practitioners urged the government to halt NHS Health Checks until’robust evidence’ existed [8]. Despite the criticisms, Public Health England relaunched the programme in 2013 with a commitment to reach an estimated 15 million eligible people in over five years, and published an implementation review and action plan to develop a programme of work to apply ‘greater rigour’ in evaluating it [4].

Current evidence suggests that those who are most at risk and potentially with most to benefit from population-level early interventions may be least likely to engage. Due to the reported lower uptake of screening in areas of higher deprivation, there are concerns that NHS Health Checks may contribute to widening inequalities in health [9–12]. Currently, people from most black and minority ethnic (BME) populations are at greater risk of diabetes and stroke compared to the majority population [13]. Health inequalities within the BME community have been attributed to institutional and socio-cultural barriers to accessing healthcare [14–16], socioeconomic inequalities and the experience of racial discrimination and harassment [17, 18].

There is evidence to suggest that community based programmes can increase uptake of preventive health initiatives such as CVD screening by capitalising on a combination of community assets, including the use of community networks, knowledge, venues, lay health workers/trainers and local media [16, 19, 20]. To help improve access, prevention programmes have been delivered outside family practices in a range of community settings including shops, pharmacies and places of work and leisure [19, 21–24]. The employment of trusted and respected community members have been found to play a key role in publicising and facilitating attendance at outreach NHS Health Checks [21] and in the delivery of health prevention interventions such as blood pressure checks, health advice and lifestyle interventions in informal, community settings [19, 25]. Outreach and health workers from similar socio-cultural backgrounds are also effective in endorsing health promotion messages and enabling people to make lifestyle changes [12, 26]. Additionally, by working with lay people to deliver health promotion programmes, a healthcare professional is more likely to gain cultural awareness of and sensitivity to the needs, values and beliefs of the target population [24]. These collaborative health partnerships are therefore more likely to deliver services which are tailored to the needs and experiences of the local community [24].

To address any potential for inequity in uptake, Bristol Public Health commissioned participating family practices to work in collaboration with health trainers from community health improvement teams. Health trainers provide community based NHS Health Checks with the aim of increasing uptake in areas of high deprivation [12].

The NHS Health Checks outreach initiative is underpinned by a community partnership model of health promotion which involves community health partners (family practices and health trainers) working with community assets to deliver outreach NHS Health Checks in community settings, including pharmacies, and community centres to ensure equity of access [2, 4]. These initiatives are set amidst a public health agenda which aims to strengthen marginalised communities, build on social capital [27] and utilise community assets to reduce inequalities [28, 29]. Community assets are defined as the individual, social, physical and environmental resources held within a community and can include: practical skills, capacity, knowledge, the passions and interests of local residents; the networks and connections in a community; public, private and third sector organisations; and the physical and economic resources within the community [30]. Increasing social capital through the use of community assets has been seen to contribute to improved community engagement in public health initiatives [16, 31], with social capital being proposed as a mediator between inequalities and health [32]. However, little is known about the value of utilising community assets to increase the engagement of a target community in outreach NHS Health Checks and the acceptability of community based outreach NHS Health checks for community residents. The present study aimed to explore the feasibility and acceptibility of utilising community assets to deliver community based NHS Health Checks targeting the Afro-Caribbean population to identify the benefits and challenges of providing NHS Health Checks via community outreach events.

## Methods

This study adopted an ethnographic approach using direct observation [33] and follow up interviews [34] with attendees and staff at outreach events in community venues in inner-city Bristol, south-west England between May 2013 and November 2013. This study was approved by the NHS Research Ethics Committee South West 4 (ref 10/H0102/39).

For each outreach event, a social scientist researcher (RR) observed: community engagement activities (e.g. the methods, location, individuals involved and types of interaction), the NHS Health Check process (e.g. how different stages of the NHS Health Check were conducted, by whom and types of interactions) and the physical environment (e.g. location of the NHS Health Checks and how they were utilised). The researcher also conducted interviews with staff and attendees. The engagement worker informed attendees about the research on arrival and the researcher provided further information about the study and gained written informed consent. Interviews were conducted in a private room at the outreach event venue directly after the NHS Health Check or in the days following in the participants’ home. Interviews explored views and experiences of the NHS Health Checks community outreach initiatives. A topic guide was used to focus the interviews (Additional file 1), however, participants were able to speak freely about their experiences and raise topics not covered by the guide. Attendees received a £10 shopping voucher as an acknowledgement of their participation. Interviews lasted between 15 and 45 minutes.

Detailed written field notes of observations were made and annotated to assist in the analysis. Semi-structured interviews were audio-recorded, transcribed, anonymised. The software NVivo10 aided data management. Analysis began alongside data collection, with ideas from early analysis informing later data collection in an iterative process. A thematic analysis [35] was used to scrutinise both the observations and interviews data involving a process of constant comparison between cases [36] in order to identify and analyse patterns and themes across the dataset. Analysis commenced with RR generating an initial coding framework, grounded in the data, which was added to and refined, with material regrouped and recoded as new data were gathered. Codes were gradually built into broader categories through comparison across transcripts and higher-level recurring themes were developed. A random sample of three transcripts were independently scrutinised against emergent coding frames by a senior social scientist (JH) to contribute to the generation and refinement of codes to maximize rigor. Emergent themes were discussed by the multi-disciplinary research team RR, JH, NC, AM, GF to ensure credibility and confirmability.

## Results

Observations were conducted of four outreach events in four different community venues in inner-city Bristol (see Table 1). Interviews were conducted with 16 outreach attendees (9 men and 7 women and aged between 42–57 years) with the majority receiving a low QRisk2 Score (see Table 2). Interviews were also conducted with four outreach staff (one family practice nurse, one health care assistant, one engagement worker, one health trainer, all female and aged between 31–48 years).

**Table 1:** Summary of NHS outreach health check events and engagement activities

Event	Venue description	Total NHS health checks conducted (*N =* 60)	Staff (*N =* 24)	Engagement workers (*N =* 8)	Target group	Time	Engagement & publicity activities
1	Church with a predominantly Afro-Caribbean congregation	23	6 (1 nurse, 1 healthcare assistant, 2 health trainers, 2 engagement workers)	1 male, 1 female from the local Black-Afro-Caribbean community	Afro Caribbean Men	10 am–2.00 pm	Peer networks and word-of-mouth; leafleting, posters, local radio and direct contact with local shops, betting shop, bar, passers- by
2	Community Centre for Rastafarians	11	6 (1 nurse, 1 healthcare assistant, 2 health trainers, 2 engagement workers)	1 male, 1 female from the local Black-Afro-Caribbean community	Afro Caribbean Men	2.00–2.30 pm	Peer networks; leafleting, posters, local radio and direct contact with local shops, taxi office, bar, passers-by
3	Community Centre	14	6 (1 nurse, 1 healthcare assistant, 2 health trainers, 2 engagement workers)	2 females from the local Black-Afro-Caribbean community	Afro Caribbean Women	1.00–6.00 pm	Leafleting, posters, local radio and direct contact with local shops, betting shop, bar, passers-by
4	Learning Centre	12	6 (1 nurse, 1 healthcare assistant, 2 health trainers, 2 engagement workers)	2 females from the local Black-Afro-Caribbean community	Afro Caribbean Men & Women	1.00–6.00 pm	Minor engagement activities prior to the event, including leafleting in local shops, a local bar, & passers-by

**Table 2:** Characteristics of interview participant attendees, *N =* 16

Characteristic	N	%
Age (years)		
40–44	5	31
45–49	4	25
50–54	3	19
55–59	4	25
Ethnic group		
Black Afro Caribbean	12	75
Black African	2	13
Mixed – White/Afro Caribbean	1	6
British Asian	1	6
Gender		
Female	7	44
Male	9	56
Cardiovascular Q Risk2 Score		
High (>20 %)	1	6
Medium (>10 % <20 %)	1	6
Low (<10 %)	12	75
Not known^a^	2	13

^a^QRisk2 calculator was not operational due to limited Wi-Fi connection

Analysis of the interview transcripts and field notes identified four main themes: community engagement; community assets; participants’ motivation for attending; experiences of NHS Health Checks.

### Observation of NHS health check outreach process

Due to the potential variation in demographic characteristics and therefore population need between different geographical areas, the Department of Health allowed substantial autonomy to local authorities in the local administration of the NHS Health Checks programme, as long as minimum standards are met [2]. In Bristol, public health commissioners responsible for NHS Health Checks set up inner-city health partnerships comprising staff from inner-city family practices, health trainers from the community health improvement teams (employed by local authority public health) and engagement workers who are residents of the local community. These health partnerships planned and delivered outreach events.

Prior to each outreach event, two engagement workers from the Afro-Caribbean community were employed to undertake pre-engagement activities such as door-to-door leafleting, contacting peers and other community members, publicising the event by distributing posters and leaflets at various amenities in the community (community centres, shops, bars, betting shop). Events one and two employed a sister/brother team to engage Afro-Caribbean men while events three and four employed two female community activists to engage the community through leafleting and other publicity techniques (see Table 1). The engagement workers for event one and two, specifically the male engagement worker capitalised on his standing in the local community to encourage his peers to attend the NHS Health Check event by phoning individuals known to him within his social network. On the day, publicity about the event was also generated through word-of-mouth recommendation which consequently generated further interest amongst his peer network. The NHS Health Check events were also publicised on the community radio.

On the day of the outreach event, a member of staff, normally an engagement worker, was stationed outside the venue to meet and greet attendees. Each components of the NHS Health Check were carried out by different staff: health trainers obtained information about the patient’s family history of CVD, lifestyle information (smoking status, physical activity, diet and alcohol consumption), body mass index (BMI) measurements, blood pressure, and cholesterol using near-patient testing equipment. At a separate station, the healthcare assistant took blood pressure and for those attendees assessed as ‘high risk’ diabetes, a glycated haemoglobin (HbA1c) blood test was conducted. At another station, nurses calculated attendees’ QRisk2 [3] result. Attendees were given written and verbal information about their blood pressure, cholesterol, BMI and QRisk2 results. Attendees were also given accompanying verbal lifestyle and health advice. Due to poor internet connections in two venues, staff were sometimes unable to access the online QRisk2 calculator and communicate the QRisk2 score to attendees.

### Community engagement

Publicity through peer groups and word-of-mouth made a significant contribution to the turnout in the first and second outreach event (*n =* 34) which targeted ‘young’ Afro-Caribbean men (aged 40–50 years). Participants indicated that it was their respect for and loyalty to the engagement worker which prompted individuals to attend a NHS Health Check:

‘Because my very good friend, [male engagement worker], called upon me. And I think if he called upon a thousand people, 999 would turn up. He’s just well-loved within the community and nobody wants to let him down. So I turned up for him’ (Attendee 4, male, 50 years, low risk)

Since many of the attendees were friends or familiar faces, publicity about the event snow-balled through opportunistically approaching peers on the street:

‘Yeah everybody who went there was like friends, so everybody was comfortable. There were people who were coming out – we were coming out of there and you see people walking past and they say, “What are you doing there?” We said, “Come in and have a chat,” and everybody was pulling people off the street too’ (Attendee 2, male, aged 42, low risk)

The combination of the engagement worker and location facilitated the uptake of NHS Health Checks. Offering NHS Health Checks in a local venue and having them endorsed by trusted community members in lay language, played a role in facilitating attendance:

‘And there was ordinary, regular people saying, “Come and have it done, ‘cos we’ve had it done, and why not?” So I think that’s what made the difference: it was in – it was in a language and in a place that the local community could understand and trust really.’ (Engagement worker)

The engagement workers also facilitated opportunistic attendance by handing out leaflets and encouraging passers-by to attend:

‘Okay, basically I was stopped in the street really with a flyer and I looked on the flyer and somebody was just letting me know a bit about the event and they said it’s good, obviously, to get a health check every year.’ (Attendee 16, male, 45 years, low risk score)

‘Well, I was just going on my way into town and the lady was outside asking people as they passed. That’s how I heard about it’ (Attendee 12, male, 43 years, low risk score)

For some, attendance was prompted by a combination of factors, such as pre-publicity on the radio combined with direct contact:

‘Well ‘cos I’ve been listening to the radio and they’ve been putting it on the radio quite a bit … to have your health check um and um, you know, that’s it’s being done in different places…Cos it’s like, you know, they can detect heart disease and stuff like that, yeah… and happened to be passing here and was told about this health check’ (Attendee 8, female, 46 years, low risk score)

Some staff indicated that some community members may have been unable to attend due to work commitments as the outreach events were held during the day:

‘A lot of that age group is people who would essentially be in work, so it’s around, you know, getting appointments’ (Health trainer).

Attendees and engagement workers identified other barriers to attendance, including a historical mistrust of external institutions or agencies amongst some community members:

“..I think in terms of the history, what happens, it feels like in all areas, whether it’s education, policing, health that the African-Caribbean community have always been on the back foot. So in terms of um a number of the men that I spoke to, a number of the people I spoke to about having a health check, it’s kind of like, “What do they want, blood?” (Engagement worker)

### Community assets

The setting of the outreach NHS Health Checks also played a key role in influencing people to attend. The NHS Health Checks were held in three community centres and a local church with a predominantly Afro-Caribbean congregation. Both participants and staff commented that the venues were both familiar and convenient due to the location:

‘So it being here [name of community centre], and because I’m always passing here, in and out of here, it was easy to just come in and do that.’ (Attendee 8, female, 46 years, low risk score)

‘Well it’s central, so it’s right in the middle of the community, so that’s something.’ (Attendee 10, female, 55 years, medium risk score)

Some participants saw the presence of local family practice staff conducting the NHS Health Checks in a community venue as an indication of family practice staff having an interest in their local community:

‘But if you – people [family practice staff] coming out to a community, then they’re coming with the – they’re showing you that they’ve got time, they’re bringing the time to come out, you know, maybe that’s better.’ (Attendee 1, male, 46 years, low risk score)

The researcher observed one attendee voicing her appreciation of Afro-Caribbean staff due to their knowledge of culturally relevant lifestyle information:

‘It’s nice to see someone from your own community there ‘cause they have a better understanding of your own culture like food and stuff’ (Female attendee)

An additional factor which facilitated attendance was the ease and expedience of the drop-in NHS Health Check which was often contrasted with some attendees’ experience of visiting the doctors:

‘It was quicker than going to the doctors actually because when you go to the doctors, you’ve got an appointment … yeah, it was very quick, very efficient’ (Attendee 11, female, 44 years, low risk score)

While the location of the NHS Health Checks were positively received, some concerns were raised about the lack of confidentiality due to the setup, size and lack of partitioning in the room in which NHS Health Checks were conducted. While events one and four used two large rooms to undertake the NHS Health Checks with a separate room employed for the discussion of the NHS Health Check results and accompanying lifestyle information, event two used one small room and a kitchen for the results and event three used one large room for all aspects of the NHS Health Check. The lack of confidentiality was identified during observations undertaken by the researcher who was able to hear confidential conversations within proximity of others attending. This was also supported by the experience of staff and attendees:

‘I don’t think um…having to sit in a kitchen angling the laptop so we got [Wi-Fi] reception…I don’t think you come across very professional when you’re sitting in a kitchen and all huddled round and all on top of each other. And it’s not very nice for the patients, because…quite personal information and everybody’s sat on top of each other. I just didn’t think it was very suitable really.’ (Practice Nurse)

‘I think um the way that that room is set up down there, it probably would be nicer to have um, I don’t know, maybe like a partition um in the room, so that like obviously when you’re being weighed and measured, do you know what I mean, you don’t – I mean how it’s set out is like everybody can see.’ (Attendee 8, female, 46 years, low risk score)

### Participants’ motivation

One of the principal motivations for attending a NHS Health Check was participants’ understanding of CVD linked to having familial CVD. Attendees talked about family members with CVD as well as diabetes, hypertension and stroke, which prompted them to attend a NHS Health Check for preventative reasons:

‘my mum’s diabetic, she’s also on kidney dialysis, which is the sort of … well they’ve put it down to the effect of being diabetic for several years, um so there is that worry um that it’s sort of in the family.’ (Attendee 9, male, 52 years, low risk score)

‘I’m over 40, um and have got a family history of the diabetes, high blood pressure and so on, so I thought it would be good for me to have the check.’ (Attendee 5, female, 57 years, low risk score)

For many of the outreach attendees the motivation to attend a NHS Health Check was linked to an interest in being proactive about their health and spoke of being health conscious and viewed the NHS Health Check as an opportunity to gain information to inform self-care strategies:

‘I do like to, you know, sometimes keep in check with how I am overall health-wise … it’s best to try to, you know, maintain a certain level of health, but you can only do that with the knowledge of how - you know, where are you, you know, in your body.’ (Attendee 1, male, 46 years, low risk score).

Some attendees used the NHS Health Check to inform themselves about lifestyle or risk factors:

‘I did put it in my diary and sort of thought it’d be good just to come and get that check. Cos sometimes I think my lifestyle could be healthier, so I do worry about, you know, whether I am actually as healthy as – as I should be.’ (Attendee 9, male, 52 years, low risk score).

Some participants were motivated to attend due to perceived concerns relating to specific risk factors such as blood pressure:

‘Yeah I thought my blood pressure might be high, (laughs) ‘cos I’ve been under a lot of stress of late. But they said oh that was fine.’ (Attendee 5, female, 57 years, low risk score)

Some participants were expecting to be tested for diabetes - perhaps because it was not clear to attendees that only attendees considered to be at risk of developing diabetes would receive a test:

‘Yeah cholesterol, yeah. I thought they were checking for diabetes actually, but they said no. So um – because I – I did check on the um leaflet. But, yeah, so they did the height, the weight, blood pressure and the cholesterol, yeah.’ (Attendee 5 female, 57 years, low risk score)

### Experience of NHS health checks

#### Benefits

The majority of people attending the observed NHS Health Check (55/60) received low CVD QRisk2 scores. Most attendees felt relieved or reassured with their low risk results which for some served to reaffirm healthy lifestyle behaviours, as these participants highlight:

‘Doing it made me feel a lot better within myself, knowing that I was alright, and going down the right road, which encourages me to still stay in my gym carrying on doing what I do.’ (Attendee 4, male, 50 years, low risk score)

Some attendees reported being motivated to make lifestyle changes after attending a NHS Health Check and appreciated being informed of what changes to make*:*

‘I was a bit high on cholesterol so, you know, therefore I adapted what I’m eating, for example, not too many saturated fats: I’ve learnt that that’s not a very good thing to be eating a lot of, you know, and er so I’ve cut down on that. And I’m trying to cut down on sugar a lot, you know, ‘cos, you know, oh you always hear sugar is not really nice. And the thing is, I’ve got a bit of a sweet tooth…it’s a bit hard for me but I try to, you know, modify that. And er, yeah, so that’s what – and blood pressure, you know, a little bit high, and things like that, so I try to then, as long as you know, then you can act on it’ (Attendee 1, male, 46 years, low risk score)

The findings also indicate that attendees valued the opportunity to talk to a health care professional about their health, to ask questions and be informed about aspects of the NHS Health Check, such as being more informed about cholesterol or blood pressure:

‘I had a good conversation with her, ‘cos I always hear people talk about cholesterol and stuff … so she was actually explaining to me about cholesterol and good cholesterol and not so good cholesterol and stuff like that, so that was good.’ (Attendee 8, female, 46 years, low risk).

‘…quite a few of them were quite chatty and wanted to know more information about their risks and, you know, what their blood pressure meant and what, you know, what the other scores meant.’ (Practice Nurse)

An additional benefit was the way in which attendees, particularly men, engaged in self-care talk through discussion with peers. In participants’ accounts and from the researcher’s observations, some men discussed their results and preparedness to make lifestyle changes with other male attendees. Some men encouraged their peers to follow up their results with their GP or make lifestyle changes:

‘It give you a bit of encouragement, say, “Yeah get along to your doctor and sort it out,” … you compare what you’re doing,..”You go to the gym?”…“What do you eat?”…“What you not eating?”…You know, “Are you drinking?” (Attendee 1, male, 46 years, low risk score).

#### Challenges

Some attendees indicated that the lifestyle advice following their results was too brief and could have been more detailed. This was particularly the case where attendees were surprised or unhappy with their test results - one attendee reported that she was “gutted” about being labelled as obese and was disappointed that she had not received more informative advice about what action they could take to improve their health:

‘Gutted, ‘cos I don’t know what else I can do… I think it is frustrating because everything now seems to be on this BMI thing… and I don’t feel that I’m obese, but they keep using that word.... Yeah I thought they would have been able to give you more – it would have been more in-depth, kind of. But I don’t know whether they [healthcare staff] haven’t got time and they felt, “OK that’s it, and then the next one,”’ (Attendee 5, female, 57 years, low risk score)

The findings also indicate that some attendees continued to hold health misconceptions after attending a NHS Health Check, for example the belief that high cholesterol can be ‘burnt off’ during exercise.

‘my blood sugar levels are nice, they said my cholesterol was a bit high and I said, that’s fine, I can burn that off, which I do, I burn it off, I sweat it out… I make my body move, I ride’. (Attendee 3, male, 49 years, low risk score)

## Discussion

This study highlights the value of community assets - local community centres, church, engagement workers, informal networks and local radio - and their role in facilitating uptake at NHS Health Check outreach events. Participants’ motivations for attending were influenced by an understanding of the putative preventative value of NHS Health Checks, often informed by their experience of familial CVD. Attendees were also motivated by a desire to be informed about risk factors and lifestyle. Highlighting the value of the prevention of CVD could therefore be used to inform future health promotion strategies to generate uptake.

There is evidence that social systems such as social capital and community assets can contribute to engagement in public health activities [16, 31] but little is known about their value in relation to community based NHS Health Checks. Social capital has been defined as “the features of social organisation such as networks, norms and trust, that facilitate coordination and cooperation for mutual benefit” [37]. In this sense, the outreach event which employed local engagement workers were viewed by many attendees as trusted and respected members of the local community who played a pivotal role in influencing people’s decision to attend a NHS Health Check. This was particularly evident in the activity of the male engagement worker who was able to co-ordinate his male peers to attend by capitalising on his status in the community. This coordination and cooperation of community members was pivotal in publicising the event and engaging people to attend, for example by directly phoning people in his social network. However, despite uptake and interest amongst the peer group of the male engagement worker, this method may have excluded people who were outside of the male engagement worker’s social network. This highlights the value of employing a range of methods to target a wide audience.

Attendees valued the convenience of local outreach events and the more relaxed, drop-in ambience of NHS Health Checks located in familiar community venues which was often contrasted with the longer waiting times and formality of family practices [22, 38–40]. Attendees also valued the presence of staff that were members of their community since they were able to discuss or provide relevant, culturally specific lifestyle information (e.g. diet). This is consistent with previous evidence highlighting that employing health workers from similar socio-cultural backgrounds to the target population was an important factor in facilitating the delivery and acceptability of community outreach events [12, 20, 40]. By working with community members to deliver health promotion programmes, a healthcare professional is more likely to gain cultural awareness and sensitivity to the needs, values, and beliefs of the target population [24]. Some participants also valued the presence of family practice staff in a community venue which signalled their interest in the health of the local community and perhaps served to overcome traditional institutional barriers [17].

Although the presence of NHS Health Checks in community venues were welcomed by many, our findings point to a number of improvements that can be made. First, organisers of community-based NHS Health Checks need to ensure venues are fit for purpose. Problems with internet connection in several of the venues prohibited staff from calculating the attendee’s cardiovascular risk, the focal point of the NHS Health Check. In addition, our findings also indicate that confidentiality may have been breached in the delivery of outreach NHS Health Checks due to the lack of appropriate space. The perception of a lack of confidentiality and fear of embarrassment around results is a major barrier to attendance at screening programmes, particularly in community venues [38]. The timing of NHS Health Checks may be a barrier to attendance for some and it is essential that outreach NHS Health Checks are provided outside working hours to maximise access. Outreach staff also need to avoid the prospect of labelling patients and communicate results and accompanying lifestyle advice in sufficient detail in order to enhance self-efficacy and enable people to make lifestyle changes. This reflects other concerns raised about the threat posed by NHS Health Checks to an individual’s autonomy and the risk of victim blaming where patients resent having their lifestyle scrutinised by the medical gaze [15]. These findings highlight the importance of ensuring staff are equipped with behavioural change techniques (such as motivational interviewing) which aim to support and enable individuals to make lifestyle changes [41].

Attending the event provided an opportunity for people to talk about their health, discuss and compare results with health workers and peers. Informal positive peer support served to endorse key health messages and played a role in encouraging some attendees with identified risk factors to make follow up appointments with their GP. The value of positive peer support in endorsing health messages and facilitating lifestyle change is supported by previous findings [25] and explained by dynamic social impact theory which posits that people are more likely to respond positively or make behaviour changes when health messages are communicated by similar or credible communicators [42]. In the future, NHS Health Check Commissioners may benefit from capitalising on community assets and health partners to overcome the types of socio-cultural barriers to attending NHS Health Checks which include use of trusted community members, use of local venues, employment of culturally identifiable staff or lay health workers to instil trust, support individuals to facilitate lifestyle change and promote culturally relevant services [16, 25, 38].

This study benefited from triangulating interviews with observational data from a range of outreach events. To maximize analytic rigour, analysis was conducted by the research team and included double-coding and consensus to arrive at the final themes. This study only reflects the views of attendees, which is a limitation of the study, and future research may therefore wish to explore those reasons for non-attendance. The views and experiences predominantly relate to participants from the Afro-Caribbean community in Bristol and may not therefore be generalisable to other BME communities.

## Conclusions

NHS Health Checks community outreach were positively received with a range of benefits conferred to those who attended. NHS Health Checks also raised awareness of the challenges and opportunities involved in bringing NHS Health Checks into a community setting. Importance needs to be given to utilising community assets such as employing local community members/organisations to engage the target community using a range of publicity methods (local ambassadors, community radio and publicity material in local venues) to maximise engagement. By bringing NHS Health Checks into the community, the traditional boundaries of healthcare provision are redefined and extended to a more localised setting. The appeal of a more localised and flexible service provided for local communities was key to facilitating attendance. Outreach events provide evidence of how local health partnerships (family practice staff and health trainers) and community assets, including informal networks, can enhance the delivery of outreach NHS Health Checks and in promoting the health of targeted communities. To deliver NHS Health Checks effectively, staff need to be provided with the appropriate training to ensure patients are supported and enabled to make lifestyle changes.

## Additional file

Additional file 1:
**Interview Topic Guide.** (DOC 38 kb)

## Abbreviations

BME: Black and minority ethnic communities
BMI: Body mass index
CVD: Cardiovascular disease
GP: General practitioner
