# Supplementary material for: The provision of NHS health checks in a community setting: an ethnographic account
Source: BMC Health Serv Res. 2015 Dec 10;15:546. doi: 10.1186/s12913-015-1209-1 (PMC4676171; doi:10.1186/s12913-015-1209-1)
Supplement: Additional file 1: — Interview Topic Guide. (DOC 38 kb) [file 12913_2015_1209_MOESM1_ESM.doc]

|  |  |
| --- | --- |

**Interview Topic Guide: Outreach Staff**

***Introduction and background***

- Background information on participant (e.g. job title, length of time practising, special interests)
- Role in outreach health checks process

# *Usual practice/ feelings about screening for* vascular risk

- Views about the value of screening people over the age of 40 for vascular risk
- Views about pros and cons of screening process
- Prior experience of treating/managing vascular risk
- Any additional training/guidance received in relation to NHS health checks

***Views and experiences of the screening process in outreach setting***

- Views about experience of delivering the checks on the day
- What worked well, What could have been done better/differently
- Views about pros and cons of screening in the community vs in practice
- Views about barriers to attending health check in GP practice vs community
- Views about the acceptability of the screening process in outreach setting for patients
- Experiences of discussing the screening results
- Decision-making about potential treatment/information options for high risk patients

***Any other issues***

- Any other issues the participant would like to raise

**Interview Topic Guide: Attendee**

***Introduction and background***

- Health is in general
- How did you initially hear about the NHS health check?, Understanding of NHS health checks?
- Why did you decide to come along for a health check? Initial feelings/expectations

***Experiences of Health Check***

- Can you tell me what happened when you had the NHS health check?
- Who carried it out and what were they like
- What information did you receive? Advice received about your health?
- How did you feel at the end of the check?
- What was helpful/useful about the appointment?
- What could have been better?
- Is there anything you would wish to change about the NHS health check?

***Any other issues***

- Any other issues the participant would like to raise
